# Ligand‑Based Virtual Screening Discovery of Thienopyrimidinone Derivatives as Allosteric nSMase2 Modulators and Antivirals Against West Nile Virus

**DOI:** 10.1002/cmdc.70452

**Published:** 2026-08-19

**Authors:** Hadrián Álvarez‐Fernández, Patricia Mingo‐Casas, Ana‐Belén Blázquez, Flavia Caridi, Miguel A. Martín‐Acebes, María‐Jesús Pérez‐Pérez, Eva‐María Priego

**Affiliations:** ^1^ Instituto de Química Médica y Biomedicina (IQMB CSIC) Madrid Spain; ^2^ Departamento de Biotecnología Instituto Nacional de Investigación y Tecnología Agraria y Alimentaria (INIA CSIC) Madrid Spain

**Keywords:** host‐directed antivirals, nSMase2, *Orthoflavivirus*, sphingolipid metabolism, thienopyrimidinones, virtual screening

## Abstract

Orthoflaviviruses such as West Nile virus (WNV), Zika virus (ZIKV), or dengue virus (DENV) represent a growing global health threat, with millions of annual infections and no approved antiviral therapies. Targeting host pathways represents an alternative antiviral strategy that may overcome limitations associated with direct‐acting antivirals, including resistance and narrow antiviral spectra. Neutral sphingomyelinase‐2 (nSMase2) plays a central role in sphingolipid metabolism and has emerged as a host factor involved in the replication of several flaviviruses including WNV and ZIKV. Herein, we report a new family of thienopyrimidinone derivatives as nSMase2 inhibitors. A ligand‐based virtual screening (LBVS) campaign using DPTIP as a template ligand identified 13 candidates from over 5 million ZINC20 compounds, which were refined through ADMET‐properties prediction and molecular docking. Experimental evaluation identified compound **16** as a novel nSMase2 inhibitor. Rational design guided by computational affinity maps enabled the synthesis of several derivatives bearing a thienopyrimidinone core. These compounds inhibited nSMase2 and showed antiviral activity against WNV in cell‑based assays. These results identify thienopyrimidinone derivatives as a new scaffold for nSMase2 inhibition and support host‐directed modulation of sphingolipid metabolism as a promising strategy for the development of broad‐spectrum antivirals against flaviviruses.

## Graphical abstract

Ligand‐based virtual screening of over five million compounds identifies a thienopyrimidinone hit as a novel allosteric nSMase2 modulator. Structure‐guided optimisation improves inhibitory potency and delivers derivatives with antiviral activity against West Nile virus, expanding the chemical space of nSMase2 inhibitors and supporting host‐directed antiviral intervention.

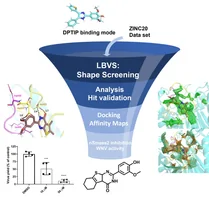

## 1 Introduction

*Orthoflavivirus* constitute a large group of emerging and re‐emerging pathogens belonging to the Flaviviridae family and include clinically important viruses such as West Nile virus (WNV), dengue virus (DENV), Zika virus (ZIKV), yellow fever virus (YFV) and Japanese encephalitis virus (JEV) [1].These mosquito‐borne viruses infect millions of individuals annually worldwide and can cause severe clinical manifestations including haemorrhagic fever, neurological disease and congenital defects [2]. Despite their global impact, therapeutic options remain extremely limited, and no specific antiviral treatment is currently approved for most orthoflaviviral infections.

Traditional antiviral development has primarily focused on direct‐acting antivirals (DAAs) targeting viral proteins involved in replication. Although this strategy has proven successful for viruses such as the human immunodeficiency virus (HIV) and hepatitis C virus (HCV), it presents several limitations, including rapid emergence of resistance and difficulties in achieving broad‐spectrum activity across different viral species. An alternative approach involves targeting host factors required for viral replication, known as host‐directed antivirals (HDAs) [3]. By interfering with cellular pathways hijacked by viruses, HDAs may provide broader antiviral activity and a higher barrier to resistance. Among host pathways involved in orthoflaviviral replication, lipid metabolism has attracted particular attention due to its essential role in viral membrane remodelling, replication complex formation and budding and release of viral particles [4, 5, 6].

**FIGURE 1:**
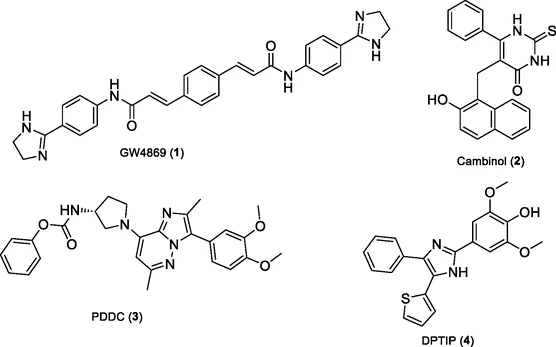
Representative nSMase2 inhibitors.

Sphingolipids represent a major class of cellular lipids involved in cellular signalling, membrane organisation and vesicular trafficking. Their metabolism has been shown to be significantly altered during flavivirus infection [7, 8, 9]. In particular, neutral sphingomyelinase‐2 (nSMase2), a plasma membrane‑associated phosphodiesterase, catalyses the hydrolysis of sphingomyelin to generate ceramide, a bioactive lipid implicated in several cellular processes including apoptosis, inflammation and membrane trafficking [10, 11]. Previous studies have demonstrated that pharmacological inhibition of nSMase2 reduces viral replication of WNV, ZIKV and DENV, validating this enzyme as a potential host‐directed antiviral target [12, 13, 14, 15, 16]. To date, only a limited number of non‐competitive or uncompetitive small‐molecule inhibitors of nSMase2 have been reported (Figure 1). Early inhibitors such as GW4869 (**1**) [17] or cambinol (**2**) [18] have been widely used as experimental tools but suffer from limited potency and selectivity. More recently, compounds such as PDDC (**3**) [19] and DPTIP (**4**) [20] have been identified as more potent inhibitors of this enzyme. Pyrazolo[1,5‐a]pyrimidine‐3,5‐diamines have been recently claimed as potent nSMase2 inhibitors in a patent highlight [21], but no further details have been disclosed.

**FIGURE 2:**
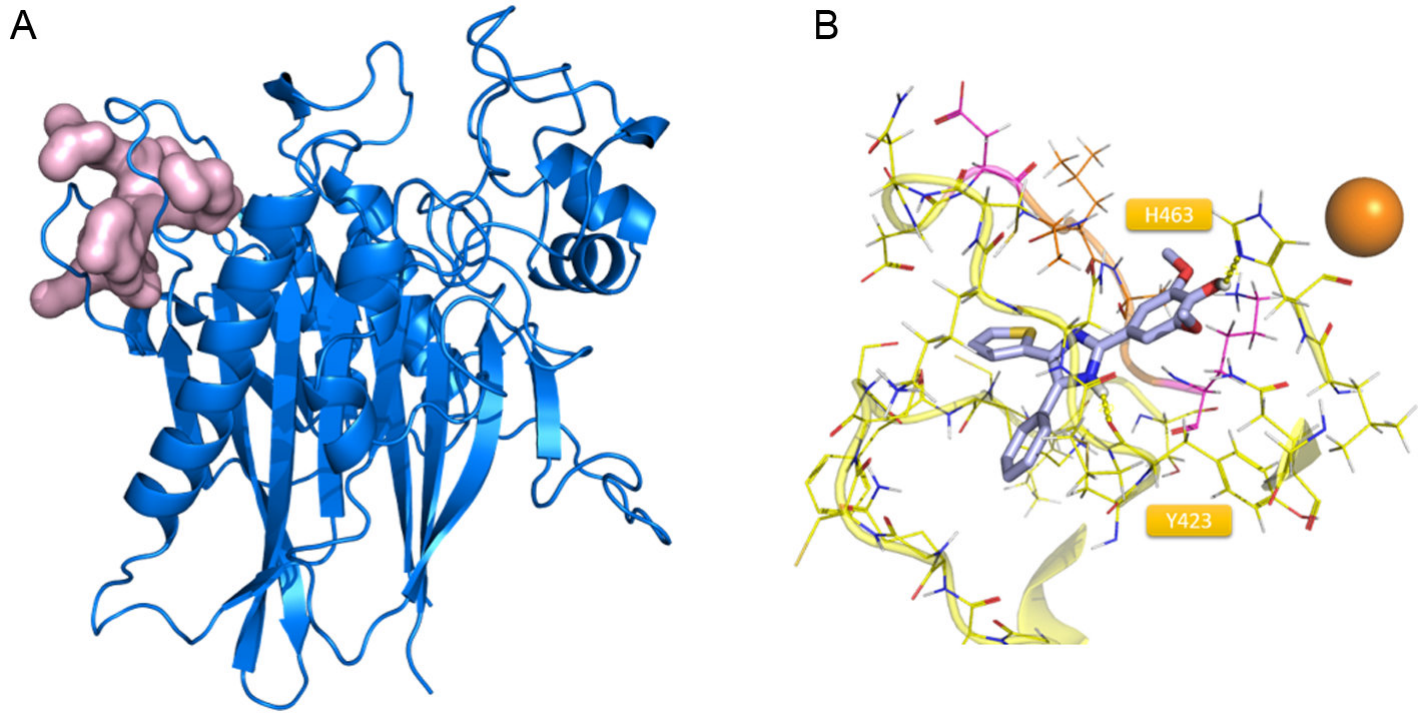
(A) Complete model of human nSMase2. The new allosteric pocket is shown in pink surface. (B) Binding mode of DPTIP (slate sticks) in nSMase2 (shown in yellow) from docking and molecular dynamics studies. Selected residues of the binding pocket are shown as yellow lines and labelled. Hydrogen bonds interactions with Tyr423 and His463 are shown as dashed lines.

We have recently reported the antiviral activity of the non‐competitive nSMase2 inhibitor DPTIP (**4**) against WNV and ZIKV [22], demonstrating potent inhibition consistent with the essential role of host sphingolipid metabolism in orthoflaviviral replication. Through extensive computational studies, we identified an unexplored allosteric pocket in nSMase2 and elucidated the binding mode of DPTIP (Figure 2), characterised by key interactions involving His463, Tyr423 and Lys465. These findings were further supported by molecular dynamics simulations and a reduced inhibition observed for the H463A nSMase2 mutant in biochemical assays. Based on this defined allosteric site and the proposed bioactive conformation of DPTIP, here we described a ligand‐based virtual screening (LBVS) strategy to identify novel chemotypes capable of allosterically modulating nSMase2 and their in vitro experimental validation.

## 2 Results and Discussion

### 2.1 Ligand‐Based Virtual Screening and Hit Identification

**FIGURE 3:**
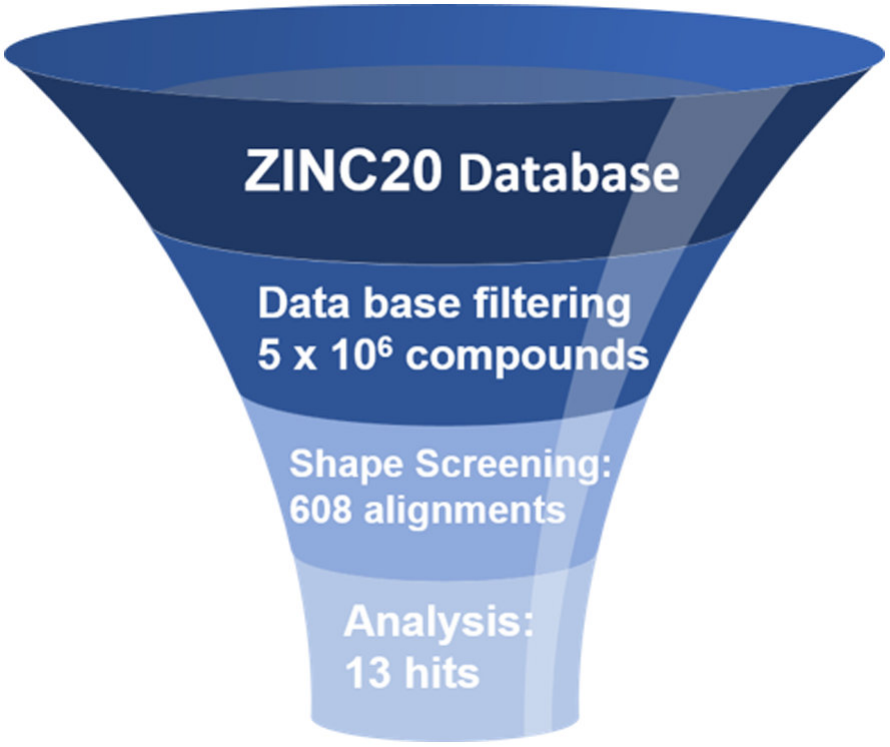
Schemtic worfflow of the ligand‐based virtual screening protocol.

To identify novel nSMase2 allosteric inhibitors, a LBVS workflow (Figure 3) was designed using the potent inhibitor DPTIP as the template ligand. Compounds used for the virtual screening campaign were obtained in SMILES format from the publicly available ZINC20 database [23]. To enrich the dataset with candidates displaying favourable physicochemical properties, lead‐like filtering criteria were applied to restrict the selection to compounds suitable for early‐stage optimisation. These filters included clogP values between −1 to 4, molecular weights ranging from 300 to 400 g/mol or PAINS analysis, which led us to a tailored dataset of approximately 5 million compounds.

Our proposed 3D bioactive conformation of DPTIP (Figure 2B) was used as template for the LBSV by means of the Shape Screening module of the Schrodinger Suite [24, 25]. Shape alignment between DPTIP and the multiple 3D conformers of the dataset, previously generated by ConfGen [26], was performed considering both steric and pharmacophoric properties. A *Similarity Score* of 0.6 was established as threshold, leading to a prioritised list of 608 candidates. A detailed visual inspection of these candidates was carried out considering structural features previously identified as key determinants for the binding of DPTIP to nSMase2. Thus, the presence of phenolic rings, hydrogen bond donor groups and the nature of the central core led us to the selection of 13 compounds, where structures, ZINC ID and similarity scores are shown in Table 1. To narrow down the number of hits to be experimentally validated, the SwissADME platform [27] was used to predict their likelihood of being P‐gp substrates. This filter helped discard compounds that may be pumped out in cell culture assays and later influence key ADME factors such as oral absorption and systemic exposure. As summarised in Table 1, compounds **6**, **10**, **12**, **13**, **16**, and **17** were predicted to behave as non‐substrates of P‐gp.

**TABLE 1:** Chemical structure of the 13 hits selected.

Compound.	ZINC ID	Sim	P‐gp substrate
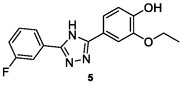	ZINC85384718	0.727	Yes
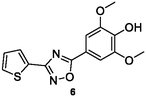	ZINC131880495	0.670	No
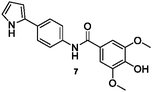	ZINC342536308	0.665	Yes
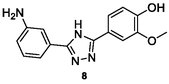	ZINC85384278	0.659	Yes
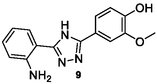	ZINC85384274	0.658	Yes
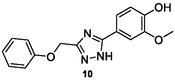	ZINC85384076	0.657	No
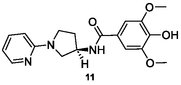	ZINC271058558	0.650	Yes
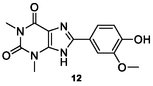	ZINC525762	0.647	No
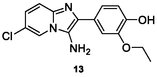	ZINC408980062	0.646	No
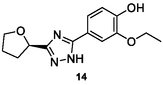	ZINC85384693	0.630	Yes
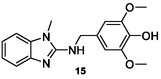	ZINC6752526	0.629	Yes
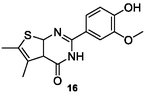	ZINC41627465	0.623	No
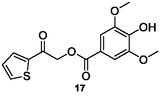	ZINC11565668	0.617	No

**FIGURE 4:**
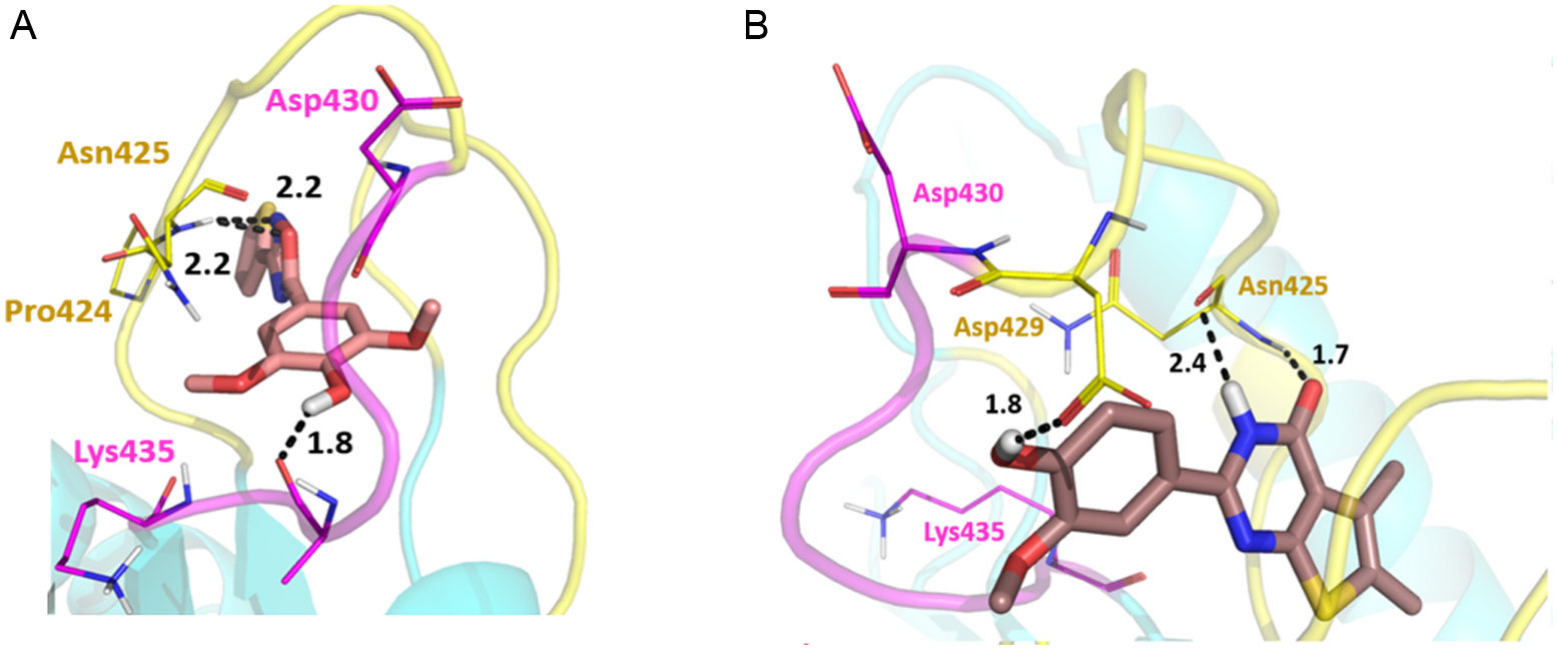
(A) Predicted binding mode of compound **6** (salmon sticks) in nSMase2. (B) Predicted binding mode of compound **16** (teal sticks). Selected residues from the binding pocket are shown as yellow and pink lines and labelled. Hydrogen bonds are shown as dashed lines.

These six hit compounds were further refined through molecular docking to explore their potential interactions with the proposed allosteric cavity for DTPIP binding within nSMase2. The best docking poses in terms of docking scores and cluster population of compounds **10**, **12**, **13**, and **17** (see Figure S1A–D) showed either steric clashes or scarce potential interactions between their respective polar groups and key residues in nSMase2, so these compounds were discarded. The best results were obtained for compounds **6** and **16**. As can be seen in Figure 4A the putative binding mode of compound **6** showed that the hydroxyl group of compound **6** could interact with the carbonyl group of Ala433. Two potential interactions were detected between the O‐1 and N‐2 atoms in the oxadiazole and the backbone NH of Asn425. Interestingly, the thiophene ring was oriented parallel to the Pro424 ring, and a putative CH‐π stacking interaction could be formed. Concerning compound **16**, the selected docking pose (Figure 4B) showed three potential hydrogen bonds: one was established between the OH of the ligand and the NH_2_ side chain of Asn429 whereas the amino and carbonyl groups of the pyrimidone scaffold could interact with the CO and NH, respectively, from the backbone of Asn425. Thus, both hits (**6** and **16**) seemed to adopt reasonable docking poses within nSMase2. However, since the final aim was to identify hits that are structurally distinct from the extensively explored DPTIP scaffold, compound **16** better fulfilled this requirement. Indeed, based on a structural similarity analysis performed using the ChemToolsHub [28] similarity calculator (Tanimoto metric), compound **6** showed a similarity score of 0.509, whereas compound **16** displayed a lower score of 0.236, indicating greater structural divergence from DPTIP for **16.**

Before testing, compound **16** was subjected to ProTox‐3.0 [29], a web‐based tool developed for the in silico prediction of toxicity using machine learning and similarity‐based methods. According to the ProTox‐3.0 (see Supporting Information), compound **16** was predicted to be inactive across all evaluated toxicity endpoints, and therefore, was selected for experimental validation.

The inhibitory activity of compound **16** against nSMase2 was evaluated using a fluorescence‐based enzymatic assay in cell lysates overexpressing nSMase2 [22]. DPTIP was used as a reference compound showing a 84% of inhibition at 10 µM, in agreement with our previously reported data in the same assay. Hit **16** showed a dose‐dependent inhibition of nSMase2 (Table 2) with approximately 71% inhibition at 50 μM and retained moderate activity at lower concentrations. Although partial inhibition was also observed in the counter assay, where nSMase2 was not present in the media, the higher inhibition detected in the primary enzymatic assay suggested that compound **16** preferentially inhibits nSMase2, supporting its selection as a novel hit compound. Structurally, **16** contains a thienopyrimidinone core that differs significantly from those of previously reported nSMase2 inhibitors. The identification of this scaffold therefore suggested the possibility of exploring new chemical spaces for the modulation of nSMase2.

**TABLE 2:** nSMase2 inhibitory activity (%) of compound 16 in cell lysates overexpressing nSMase2 (*n *= 3) and counter assay with phosphorylcholine (*n* = 3).

Concentration, µM	% inhibition nSMase2	% Inhibition counter screen	Ratio %nSMase2 inhibition/%inhibition counter screen
100	78	38	2.02
50	71	27	2.65
25	52	20	2.61
12.5	48	14	2.61

### 2.2 Rational Design of Hit 16 Derivatives

**FIGURE 5:**
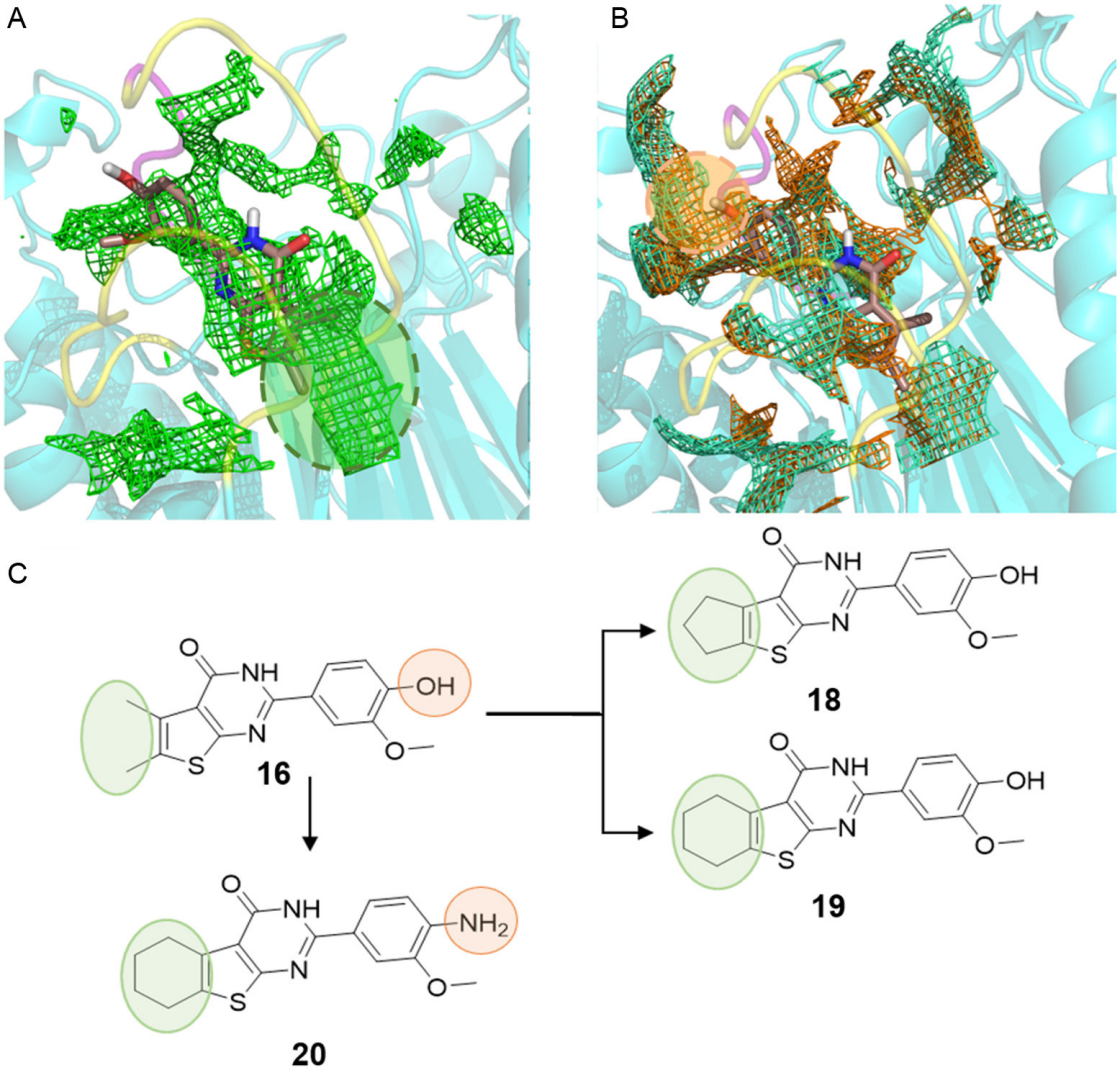
Design of new derivatives based on hit **16**. (A) Contour maps generated by cGRILL for a hydrophobic probe. (B) Contour maps for the hydrongen‐bond donor (teal) and mixed H‐bond aceptor/donor (orange) probes. (C) Chemical structures of the new derivatives.

To improve the inhibitory potency of the hit compound, a rational design strategy guided by cGRILL‑derived affinity maps was applied [30, 31]. The analysis identified two regions within the predicted allosteric binding site occupied by **16** that offered opportunities to introduce new interactions expected to reinforce target engagement. In particular, using the aliphatic probe (green mesh in Figure 5A), a hydrophobic region adjacent to the dimethylthiophene moiety of **16** was identified. This region appeared suitable for expansion through the incorporation of aliphatic rings capable of increasing hydrophobic interactions with the protein surface. Thus, derivatives containing cyclopentyl‐ and cyclohexyl‐fused rings were designed, leading to compounds **18** and **19**, respectively (Figure 5C).

Additionally, affinity mapping using the hydrogen‐bond donor (teal mesh in Figure 5B) and the mixed hydrogen bond donor/acceptor (orange mesh in Figure 5B) probes indicated the presence of a favourable region surrounding the phenolic hydroxyl group of hit **16**. To investigate the role of this interaction, the hydroxyl group was replaced by an amino group as in compound **20** (Figure 5C).

### 2.3 Synthesis of the New Thienopyrimidinone Derivatives

**SCHEME 1:**
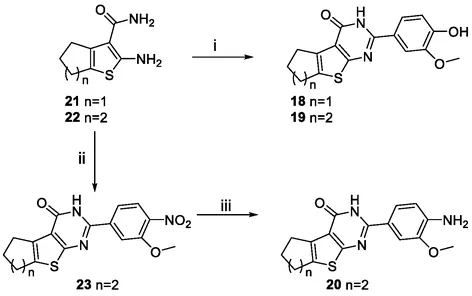
Reagents and conditions: (i) vanillin, cat HCl, n‐BuOH, 80 °C, overnight. (ii) I_2_, ACN, 3‐methoxy‐4‐nitrobenzaldehyde, rt, 1 h. (iii) SnCl_2,_ cat HCl, EtOH/EtOAc, reflux, 2 h.

The synthesis of the proposed derivatives **18–20** was carried out following the synthetic route shown in Scheme 1.

Compounds **21** and **22** were obtained by reaction of the corresponding cyclic ketone with cyanoacetamide and sulphur in the presence of morpholine in ethanol as described [32, 33]. Further reaction of **21** and **22** with 4‐hydroxy‐3‐methoxybenzaldehyde in n‐butanol at 80 °C in the presence of catalytic amounts of HCl provided compounds **18** and **19**. Additionally, compound **22** was treated with 3‐methoxy‐4‐nitrobenzaldehyde in acetonitrile in the presence of iodine, as described for similar thienopyrimidinones [34], to provide the nitro derivative **23**. Reduction of **23** by treatment with SnCl_2_ in EtOH/EtOAc in the presence of concentrated HCl at reflux afforded compound **20**.

### 2.4 Inhibition of nSMase2 Activity and Antiviral Evaluation Against West Nile Virus of 18‐20

The inhibitory activity of the synthesised derivatives (**18‐20**) against nSMase2 was evaluated using the fluorescence‐based assay described above employing cell lysates overexpressing nSMase2. Interferences with the detection cascade were assessed using the corresponding counter assay. Compounds were tested at concentrations ranging from 25 to 7.5 μM, and their inhibitory activity was compared with that of the parent compound **16** (Table 3).

**TABLE 3:** Inhibitory activity (%) of compounds 18‐20 against nSMase2 in cell lysates overexpressing nSMase2 (*n* = 3) and in the counter assay with phosphorylcholine (*n* = 3).

Comp.	% Inhibition							
	25 µM		12.5 µM		10 µM		7.5 µM	
	nSMase^a^	CA^b^	nSMase^a^	CA^b^	nSMase^a^	CA^b^	nSMase2^a^	CA^b^
**18**	81	4	61	11	77	7	70	9
**19**	90	72	83	28	69	24	71	17
**20**	77	4	59	1	56	4	22	1
**16**	52	20	48	14	n.d.^c^	n.d.^c^	n.d.^c^	n.d.^c^

a nSMase2 cell lysate.

b CA = counter assay.

c n.d. = not determined.

All new derivatives displayed improved inhibition compared to **16**. At 25 μM, **16** showed 52% inhibition of nSMase2 activity, whereas the new compounds showed inhibition values ranging from 77% to 90%. Notably, compounds **18** and **19** retained substantial inhibitory activity at lower concentrations. At 7.5 μM, these derivatives retained about 70% inhibitory activity, while compound **20** displayed a markedly lower level of inhibition.

These results clearly indicate that the incorporation of fused aliphatic rings into the thiophene moiety significantly enhances the inhibitory potency. In contrast, replacement of the phenolic hydroxyl group (compounds **18** and **19**) by an amino group (compound **20**) led to a noticeable reduction in potency, suggesting an important role for this hydroxyl group. Moreover, all compounds showed reduced inhibition in the counter assay relative to the primary enzymatic assay, indicating only limited interference with the coupled detection system and supporting direct inhibition of nSMase2.

**FIGURE 6:**
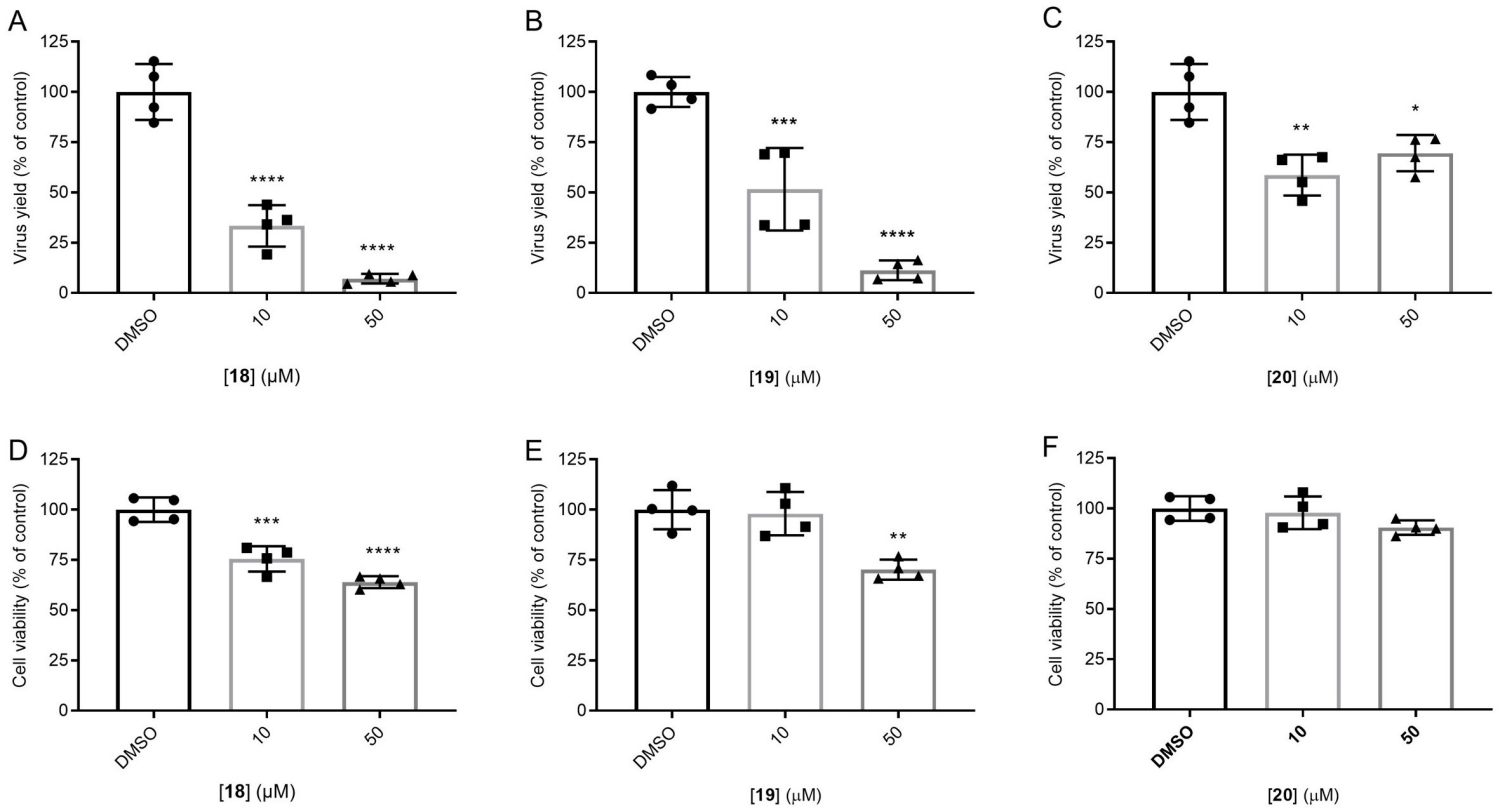
Antiviral activity against WNV and cytotoxicity evaluation of compounds **18–20** at two concentrations. (A–C) Antiviral activity. (A) Compound **18**. (B) Compound **19**. (C) Compound **20**. (D–F) Cytotoxicity evaluation. (D) Compound **18**. (E) Compound **19**. (F) Compound **20**. Bars indicate mean ± SD. Each symbol denotes an independent biological replicate (*n* = 4). ** for *P* < 0.005; *** for *P* < 0.0005; **** for *P* < 0.0001 for Dunnet’s multiple comparison tests between DMSO and treated samples performed with GraphPad Prism 8.

Next, the antiviral activity of compounds **18‐20** was evaluated in cell‐based assays against West Nile virus (WNV) using Vero cells at two concentrations (50 and 10 μM). This cell line was selected as it constituted a widely used platform for *orthoflavivirus* propragation which carries a homologous gene of human nSMase2 (accession NW_023666047.1) sharing 98.6% amino acid identity with human enzyme and whose expression has been previously verified by transcriptomics (Gene Expression Omnibus accession GSE309915). All tested compounds reduced viral replication relative to the DMSO control, indicating that inhibition of nSMase2 can effectively interfere with the viral lifecycle (Figure 6). Among the tested molecules, compound **18** (Figure 6A) showed the strongest antiviral activity at both concentrations tested. Compound **19** (Figure 6B) also showed good antiviral effects, although the viral inhibition observed at 10 μM was lower than that of **18.** In contrast, compound **20** (Figure 6C) exhibited weaker and non‐dose‐dependent antiviral activity, consistent with its lower potency in the enzymatic assay. It should be noted that hit **16** did not show antiviral activity against WNV even at 100 µM (data not shown).

Cytotoxicity was evaluated in parallel in Vero cells. Compound **18** (Figure 6D) showed significant cytotoxicity at the highest concentration tested (50 μM). In contrast, compound **19** (Figure 6E) displayed only moderate cytotoxicity at 50 μM and no detectable toxicity at 10 μM, while compound **20** (Figure 6F) showed negligible cytotoxicity across the tested concentrations.

Considering both antiviral activity and cell viability, compound **19** provided the most favourable pharmacological profile with lower cytotoxicity and a clear antiviral activity. Under the same experimental conditions in Vero cells, DPTIP exhibited a half‐maximal effective concentration (EC_50_) of 0.26 µM against WNV and a half‐maximal cytotoxic concentration (CC_50_) of 54.83 µM. In comparison, compound 19 showed cytotoxicity within a similar range to that reported for DPTIP. However, its antiviral potency appeared to be lower, since a 50% reduction in virus yield was achieved at a concentration of 10 µM.

## 3 Conclusion

A ligand‐based virtual screening campaign, combined with ADMET properties prediction and molecular docking, has led us to the discovery of hit **16**, a novel nSMase2 inhibitor containing a thienopyrimidinone scaffold. This initial hit provided a promising starting point for chemical exploration. Structure‐guided optimisation has enabled the synthesis of several derivatives with significantly improved inhibitory potency against nSMase2. This improvement in nSMase2 inhibition was accompanied by significant antiviral activity against WNV, antiviral activity that was not observed for the hit compound **16**. Among the here described compounds, compound **19** displayed the most favourable profile and was selected as the lead compound of this series.

The identification of thienopyrimidinone‐based inhibitors expands the chemical space of nSMase2 modulators and further supports host‐directed targeting of sphingolipid metabolism as a promising strategy for antiviral drug development. Although nSMase2 inhibition is not expected to completely abolish viral replication, it can substantially reduce viral burden and limit virus dissemination, making this approach particularly attractive for combination therapies with direct‐acting antivirals. In this regard, thienopyrimidinone inhibitors represent a novel scaffold warranting further investigation and contribute to the growing repertoire of nSMase2 inhibitors. Future work will focus on the optimisation of this chemical series to improve potency, selectivity, and therapeutic window, as well as on the evaluation of its antiviral activity against additional orthoflaviviruses.

## 4 Experimental Section

### 4.1 Computational Methods

#### 4.1.1 Ligand‐Based Virtual Screening

##### 4.1.1.1 Dataset

To optimise the screening process, various filters were applied to the ZINC database [23] to narrow down the list of screening candidates: Compounds had to be in stock, molecular weight was restricted to a range between 300 and 450 g/mol, compound charge was limited to + 1/−1. Ionisable compounds would be in the predominant form for a set pH of 7.4. ClogP values were set between −1 and 4 and compounds containing PAINS groups were discarded to avoid non‐specific interactions. A final data set of 5,154,048 was obtained.

##### 4.1.1.2 Shape Screening

For shape‐based screening, Shape Screening tool in the Schrödinger Suite was employed [25]. The shape similarity parameter was set to the pharm option. DPTIP binding mode was used as the query structure. Conformers for dataset compounds were generated on the fly using the ConfGen168 conformational search. A maximum of 100 conformers was generated for each compound. A filter was set to keep alignments with a similarity score greater than or equal to 0.6. From a total of 608 alignments, 13 compounds were selected for a detailed study.

#### 4.1.2 Docking Studies

Docking studies were carried out using AutoDock 4.2 [35]. The pdbqt files for nSMase2 and compounds **6**, **10–** **13**, **16**, and **17** were generated using AutoDock Tools 1.5.6. A cubic grid measuring 45 × 45 × 45 points with a spacing of 0.375 Å was established, encompassing residues from the palmitoylation loop, the DK switch loop, His463 and Gln475. AutoGrid 4.2.6 was used to calculate electrostatic, desolvation, and affinity maps for the atom types in the compounds. The Lamarckian genetic algorithm in AutoDock 4.2 was utilised to create docked conformations within the proposed binding site by randomly altering the molecule’s orientation and the torsion angles of all rotatable bonds. Default settings were maintained, except for the number of runs, population size, and maximum energy evaluations, which were set at 250, 100, and 250,000, respectively. Rapid energy evaluations for each configuration, both intra‐ and intermolecular, were achieved by pre‐calculating the receptor’s atomic affinity potentials for aliphatic and aromatic carbon, oxygen, nitrogen and hydrogen atoms within the three‐dimensional grid. A distance‐dependent dielectric function was applied to calculate electrostatic interactions. The 250 poses generated were analysed, clustered and ranked according to the binding energies calculated by AutoDock.

#### 4.1.3 Affinity Maps

Binding pocket analysis of the allosteric binding site was carried out with cGRILL [30, 31], a computational tool formally equivalent to Goodford´s program GRID [36]. The complex structure was submitted to the H++ server [37], to generate pqr format files, which included hydrogen atoms, atom point charges, and radii for all atoms. Grid centre was defined by selecting the approximate centre of compound **16** in the selected pose. A cubic box with dimensions of 20 × 20 × 20 points and a grid spacing of 0.5 Å was configured for the calculations. At each grid point, cGRILL calculated the interaction energy between the receptor and five distinct probes, incorporating terms for van der Waals (Lennard‐Jones potential), electrostatic (Coulombic), and geometry‐based hydrogen bonding [38].

### 4.2 Biological Methods

#### 4.2.1 Fluorescence‐Based nSMase2 Activity Assay

Recombinant nSMase2 (SMPD3) production and nSMase2 activity evaluation was carried out following the described procedure [22]. All the reagents necessary for the fluorescence measurement were included in the Amplex Red Sphingomyelinase Kit (ThermoFisher Scientific, Waltham, MA, USA). The assay was carried out in black solid bottom, 96‐well plates (ThermoFisher, Nunc F96), with a final volume of 200 µL, using the Amplex Red reagent as described before [18]. Briefly, using a protein concentration of 2 µg/mL, the enzymatic activity was assessed by monitoring the conversion of Amplex Red to resorufin (excitation/emission: 530/590 nm). Test compounds were preincubated with the enzyme at 37 °C for 15 min prior to the addition of a reaction mixture containing Amplex Red (50 µM), sphingomyelin (SM, 0.25 mM), and coupling enzymes (alkaline phosphatase, 4 U/mL; choline oxidase, 0.1 U/mL; and horseradish peroxidase, 1 U/mL) in reaction buffer (0.1 M Tris‐HCl, 10 mM MgCl_2_, pH 7.4, 0.2% Triton X‐100). The reaction mixtures were then incubated for an additional 1 h at 37 °C. Resorufin production was quantified by measuring relative fluorescence units (RFU). The percentage of inhibition was determined relative to the control reaction performed in the absence of inhibitor. Relative enzyme activity was calculated by dividing the change in fluorescence intensity (FI) observed in the presence of inhibitor by the corresponding FI change in the control sample. The percentage inhibition was then obtained as: % Inhibition = 100 − Relative Activity (%).

The counter assay (CA) was performed following the same protocol as the nSMase2 assay, with two modifications. First, the Amplex Red reaction mixture was prepared without sphingomyelin while maintaining all other component concentrations unchanged. Second, the nSMase2 cell lysate was replaced with a phosphocholine (PC) solution at a final concentration of 2 µM. Test compounds were preincubated with PC at 37 °C before addition of the Amplex Red mixture, followed by a further 1 h incubation. Inhibition values in the counter assay were calculated using the same procedure described for the nSMase2 assay.

#### 4.2.2 Antiviral Assay

Infectious virus manipulations were performed in BSL‐3 facilities on Vero cells (ATCC Cat# CCL‐81.4, RRID:CVCL_0059). The origin of WNV New York 99 and American ZIKV PA259459 has been previously described [39]. Infections in liquid medium and virus titrations in semisolid agar medium were performed as described [40, 41]. Briefly, to determine the antiviral activity of the compounds, Vero cells cultured in Minimum Essential Medium Eagle (MEM, Corning, NY, USA) were infected with WNV at a multiplicity of infection (MOI) of 1 plaque forming unit (PFU)/cell. After 1 h of adsorption at 37 °C, viral inoculum was removed, and fresh medium supplemented with 1% fetal bovine serum and containing the compounds was added. Virus titres were determined 24 h post‐infection by standard plaque assay in semisolid agar medium. The cytotoxicity of the compounds was determined in parallel in uninfected cultures by quantification of cellular ATP using CellTiter‐Glo luminescent cell viability assay (Promega, Madison, WI, USA).

#### 4.2.3 Statistics

For cell viability and antiviral activity, data were expressed as percentage of control DMSO‐ treated samples. Results were expressed as mean ± standard deviation (SD), with each symbol representing one independent biological replicate (*n* = 4). Statistical analyses were performed using GraphPad Prism version 8 (GraphPad Software, San Diego, CA, USA). Differences between DMSO‐treated controls and compound‐treated samples were evaluated by ordinary one‐way analysis of variance (ANOVA), followed by Dunnett’s multiple comparisons test (two‐tailed). Asteriks in the figures denote adjusted *p* values: ** for *p* < 0.005; *** for *p* < 0.0005; **** for *p* < 0.0001.

### 4.3 Chemistry

Melting points were measured on a M170 apparatus (Mettler Toledo, Columbus, Ohio, USA) apparatus and are uncorrected. The elemental analysis was performed with a CHN‐O‐RAPID instrument (Heraus, Hanau, Germany). The elemental compositions of the compounds agreed within ±0.4% of the calculated values.

^1^H and ^13^C NMR spectra were recorded on a Varian INNOVA (now Agilent, Santa Clara, CA, USA) a Varian INNOVA‐400 operating at 399 MHZ (^1^H) and 99 MHz (^13^C), respectively, and a VARIAN SYSTEM‐500 operating at 499 MHz (^1^H) and 125 MHz (^13^C), respectively. Monodimensional ^1^H and ^13^C spectra were obtained using standard conditions.

Compounds were analysed by HPLC/MS with a e2695 LC (Waters, Milford, Massachusetts, USA), coupled to a Waters 2996 photodiode array detector and a Waters Micromass ZQ. The column used is a Waters SunFire C18 2.1 × 50 mm, 3.5 µm, and the mobile phases were acetonitrile and H_2_O, together with a constant 5% of H_2_O with 2% formic acid. For high‐resolution mass spectrometry (HRMS), an Agilent 6520 accurate mass quadrupole time‐of‐flight (QTOF) platform coupled with LC/MS and equipped with an electrospray interface (ESI) working in the positive‐ion (ESI+) and negative‐ion (ESI−) modes was used.

The conversion of starting material to reaction products was followed by HPLC analysis performed in Agilent 1120 compact LC, column ACE 5 C18−300 (15 cm × 4.6 mm), UV detection was performed at *λ* = 254 nm, and the flow rate was 1mL/min, using as mobile phase A H_2_O and as mobile phase B CH_3_CN (both containing 0.05% TFA).

Analytical TLC was performed on silica gel 60 F254 (Merck, Dramstand, Germany)‐precoated plates (0.2 mm). Spots were detected under UV light (254 nm) and/or charring with ninhydrin or phosphomolybdic acid.

Flash chromatography on a Biotage Selekt system with cartridges of silica gel Biotage Sfär Silica HC Duo 20 mm and Biotage Sfär C18 Duo 30 mm.

#### 4.3.1 2‐(4‐hydroxy‐3‐methoxyphenyl)−3,5,6,7‐tetrahydro‐4*H*‐cyclopenta [4, 5]thieno[2,3‐*d*]pyrimidin‐4‐one (18)

To a solution of **21** [32] (0.16 g, 0.85 mmol) and vanillin (0.11 g, 0.72 mmol) in n‐BuOH (10 mL), concentrated HCl (0.15 mL) was added. The reaction was left stirring at 80 °C overnight. Then, the precipitated solid was filtrated and washed with H_2_O. **18** (0.04 g, 16%) was obtained after flash chromatography (DCM/MeOH 0.5% –2%). M*p* > 250 °C ^1^H NMR (500 MHz, DMSO‐d_6_) *δ*: 12.37 (s, 1H), 9.76 (s, 1H), 7.73 (d, *J* = 2.1 Hz), 7.67 (dd, *J* = 2.1, 8.4 Hz, 1H), 6.87 (d, *J* = 8.4 Hz), 3.87 (s, 3H), 2.94−2.90 (m, 4H), 2.40−2.37 (m, 2H), 1.83−1.76 (m, 4H); ^13^C NMR (125 MHz, DMSO‐d_6_) *δ*: 169.3, 159.1, 152.3, 150.4, 148.0, 140.0, 137.3, 123.1, 121.8, 118.2, 115.9, 111.6, 56.2, 29.5, 29.2, 27.9. HRMS (QTOF) m/z calculated for C_16_H_14_N_2_O_3_S: 314.0725, found: 314.0719.

#### 4.3.2 2‐(4‐hydroxy‐3‐methoxyphenyl)−5,6,7,8‐tetrahydrobenzo [4, 5]thieno[2,3‐*d*]pyrimidin‐4(3*H*)‐one (19)

To a solution of **22** [33] (0.32 g, 1.61 mmol) and vanillin (0.16 g, 1.07 mmol) in n‐BuOH (10 mL) concentrated HCl (0.16 mL) was added. The reaction was left stirring at 80 °C overnight. After completion, the solid in suspension was filtrated, washed with H_2_O and lyophilised. **19** (0.12 g, 34%) was obtained as a brown solid. M*p* > 250 °C. ^1^H NMR (500 MHz, DMSO‐d_6_) δ: 12.29 (s, 1H), 9.75 (s, 1H), 7.73 (d, *J* = 2.1 Hz), 7.67 (dd, *J* = 2.1, 8.4 Hz, 1H), 6.87 (d, *J* = 8.4 Hz), 3.87 (s, 3H), 2.90 (t, *J* = 5.5 Hz, 2H), 2.74 (t, *J* = 5.5 Hz, 2H), 1.83−1.76 (m, 4H); ^13^C NMR (125 MHz, DMSO‐d_6_) δ: 163.9, 159.4, 152.4, 150.4, 147.6, 131.9, 131.2, 123.1, 121.8, 120.5, 115.9, 111.5, 56.2, 25.8, 25.0, 23.0, 22.6. HRMS (QTOF) m/z calculated for C_17_H_16_N_2_O_3_S: 328.0882, found: 328.0874. The spectroscopic data are consistent with those previously reported [33].

#### 4.3.3 2‐(3‐methoxy‐4‐nitrophenyl)−5,6,7,8‐tetrahydrobenzo [4, 5]thieno[2,3‐d]pyrimidin‐4(3*H*)‐one (23)

To a solution of **22** (0.30 g, 1.55 mmol) and 3‐methoxy‐4‐nitrobenzaldehyde (0.34 g, 1.86 mmol) in acetonitrile (10 mL), molecular iodine was added (0.51 g, 2.01 mmol). Reaction was monitored by TLC until completion. It was quenched using a 5% aqueous solution of Na_2_S_2_O_3_, filtered and the solid was washed with Et_2_O (x3). **23** was obtained as a yellow solid (0.17 g, 31% yield). M*p* > 250 °C. ^1^H NMR (500 MHz, DMSO‐d_6_) *δ*: 12.78 (s, 1H), 8.02 (d, *J* = 1.5 Hz, 1H), 8.00 (d, *J* = 8.5 Hz, 1H), 7.85 (dd, *J* = 1.6, 8.5 Hz, 1H), 4.04 (s, 3H), 2.92 (t, *J* = 5.9 Hz, 2H), 2.78 (t, *J* = 5.8 Hz, 2H), 1.83−1.77 (m, 4H); ^13^C NMR (125 MHz, DMSO‐d_6_) *δ*: 162.9, 159.1, 152.3, 150.5, 140.8, 137.5, 134.3, 131.5, 125.7, 122.06, 120.3, 113.8, 57.5, 27.7, 25.1, 22.9, 22.2. HRMS (QTOF) m/z calculated for C_17_H_15_N_3_O_4_S: 357.0783, found: 357.0776.

#### 4.3.4 2‐(4‐amino‐3‐methoxyphenyl)−5,6,7,8‐tetrahydrobenzo [4, 5]thieno[2,3‐*d*]pyrimidin‐4(3*H*)‐one (20)

To a suspension of **23** (0.13 g, 0.35 mmol) and SnCl_2_ in EtOH/EtOAc (1:1, 6 mL) concentrated HCl was added (0.2 mL). The reaction was refluxed for 2 h and left to cool down to room temperature. Then, 10 mL of EtOAc were added. The aqueous and organic layers were stirred with celite, and filtered. The organic phase was washed with a NaHCO_3_ solution, dried over anhydrous Na_2_SO_4_ and evaporated. The resulting solid was washed with a solution of MeOH/Et_3_N. **20** was obtained as a yellow solid (0.07 g, 66% yield). M*p* > 250 °C. ^1^H NMR (500 MHz, DMSO‐d_6_) *δ*: 12.07 (s, 1H), 7.63−7.61 (m, 2H), 6.67 (d, *J* = 8.1 Hz, 1H), 5.49 (s, 2H), 3.86 (s, 3H), 2.88 (t, *J* = 5.5 Hz, 2H), 2.72 (t, *J* = 5.5 Hz, 2H), 1.82−1.75 (m, 4H); ^13^C NMR (125 MHz, DMSO‐d_6_) *δ*: 164.3, 159.5, 152.9, 146.0, 142.1, 131.1, 131.0, 122.3, 119.9, 118.7, 113.1, 109.7, 56.0, 25.8, 25.0, 23.0, 22.3. HRMS (QTOF) m/z calculated for C_17_H_17_N_3_O_2_S: 327.1041, found: 327.1038.

## Funding

This study was supported by MCIN/AEI (PID2022–137372ORC22, PID2022–137372ORC21).

## Conflicts of Interest

The authors declare no conflicts of interest.

## Supporting information

Supplementary Material

## Data Availability

The data that support the findings of this study are available from the corresponding author upon reasonable request.
