# Supplementary material for: Ligand‑Based Virtual Screening Discovery of Thienopyrimidinone Derivatives as Allosteric nSMase2 Modulators and Antivirals Against West Nile Virus
Source: ChemMedChem. 2026 Aug 19;21(16):e70452. doi: 10.1002/cmdc.70452 (PMC13490627; doi:10.1002/cmdc.70452)
Supplement: Supplementary file 1 [file CMDC-21-e70452-s001.pdf]

Supplementary material related to the article:

Ligand-Based Virtual Screening Discovery of Thienopyrimidinone Derivatives as Allosteric nSMase2 Modulators and Antivirals Against West Nile Virus

Hadrián Álvarez-Fernández,[a] Patricia Mingo-Casas,[b] Ana-Belén Blazquez,[b] Flavia Caridi,[b] Miguel A Martín-Acebes,[b] María-Jesús Pérez-Pérez [a] and Eva-Maria Priego[a*]

[a]Instituto de Química Médica (IQM, CSIC) Juan de la Cierva 3, 28006 Madrid, Spain

[b]Departamento de Biotecnología, Instituto Nacional de Investigación y Tecnología Agraria y Alimentaria, (INIA, CSIC), 28040 Madrid, Spain

Includes:

Figure S1**.** Docking poses of compounds **10, 12, 13** and **17** S2

Figure S2. ProTox-3.0 prediction for compound **16** S2

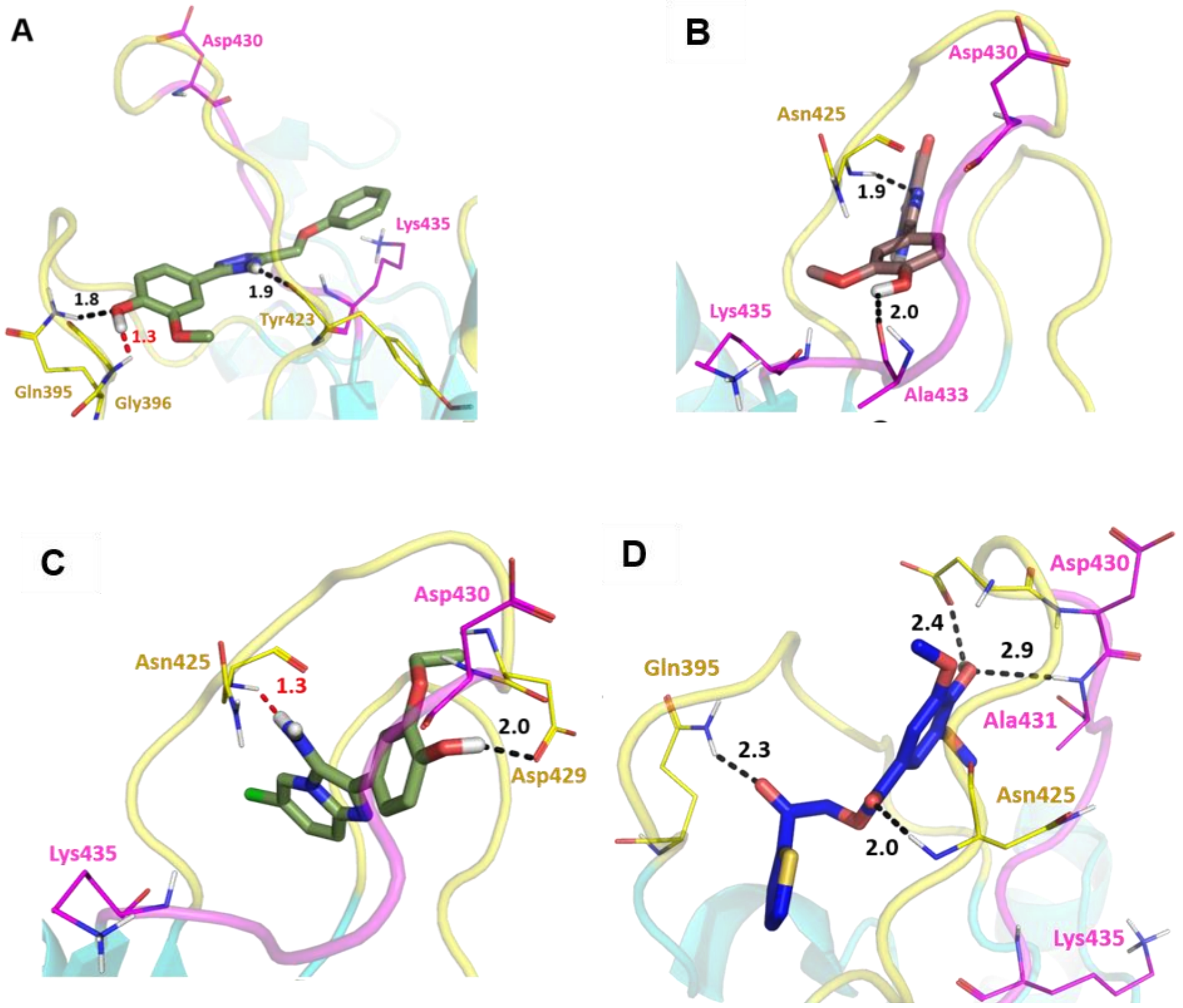

Figure S1. Docking poses of compounds **10** (A), **12**(B), **13** (C) and **17**(D)

Hit 16

ProTox-3.0 - Prediction of TOXicity of chemicals

Classification	Target	Shorthand	Prediction	Probability
Organ toxicity	Hepatotoxicity	dili	Inactive	0.50
Organ toxicity	Neurotoxicity	neuro	Inactive	0.57
Organ toxicity	Nephrotoxicity	nephro	Inactive	0.55
Organ toxicity	Cardiotoxicity	cardio	Inactive	0.72
Toxicity end points	Carcinogenicity	carcino	Inactive	0.52
Toxicity end points	Mutagenicity	mutagen	Inactive	0.57
Toxicity end points	Cytotoxicity	cyto	Inactive	0.63

Figure S2. ProTox-3.0 prediction for compound **16**
